# Crystal structure of an engineered YopM-InlB hybrid protein

**DOI:** 10.1186/1472-6807-14-12

**Published:** 2014-03-27

**Authors:** Dennis Breitsprecher, Ermanno Gherardi, Willem M Bleymüller, Hartmut H Niemann

**Affiliations:** 1 Department of Molecular Structural Biology, Helmholtz Centre for Infection Research, 38124 Braunschweig, Germany; 2 Unit of Immunology and General Pathology, Department of Molecular Medicine, University of Pavia, 27100 Pavia, Italy; 3 Department of Chemistry, Bielefeld University, PO Box 10 01 31, 33501 Bielefeld, Germany; 4 Center for Biotechnology (CeBiTec), Bielefeld University, 33501 Bielefeld, Germany

**Keywords:** Capping structure, Cap domain, Chimeric protein, Hybrid protein, Internalin, Leucine-rich repeat, LRR, Protein chimera, Protein engineering, Protein stability

## Abstract

**Background:**

The multi-domain protein InlB (internalin B) from *Listeria monocytogenes* is an agonist of the human receptor tyrosine kinase MET. Only the internalin domain directly interacts with MET. The internalin domain consists of seven central leucine-rich repeats (LRRs) flanked by an N-terminal helical cap domain and a C-terminal immunoglobulin-like structure. A potential function of the N-terminal cap in receptor binding could so far not be demonstrated by deleting the cap, since the cap is also implicated in nucleating folding of the LRR domain.

**Results:**

We generated an InlB variant (YopM-InlB) in which the InlB cap domain was replaced by the unrelated N-terminal capping structure of the LRR protein YopM from *Yersinia enterocolitica*. The crystal structure of the engineered protein shows that it folds properly. Because the first LRR is structurally closely linked to the cap domain, we exchanged LRR1 along with the cap domain. This resulted in unexpected structural changes extending to LRR2 and LRR3, which are deeply involved in MET binding. As a consequence, the binding of YopM-InlB to MET was substantially weaker than that of wild type InlB. The engineered protein was about one order of magnitude less active in colony scatter assays than wild type InlB.

**Conclusions:**

We obtained a well-behaved InlB variant with an altered N-terminal capping structure through protein design. The reduced affinity for MET precludes a straightforward interpretation of the results from cell-based assays. Still, the engineered hybrid protein induced cell scatter, suggesting that the cap is required for folding and stability of InlB but is not essential for interactions that assemble the signalling-active receptor complex. The cap swap approach described here is clearly applicable to other *L. monocytogenes* internalins and other LRR proteins such as YopM and may yield useful structure/function correlates within this protein family.

## Background

InlB is a surface-associated or secreted protein that mediates uptake of the pathogen *Listeria monocytogenes* into normally non-phagocytic cells by specifically stimulating the receptor tyrosine kinase MET on host cells [1-4]. InlB consists of an internalin domain, a B-repeat and three GW domains [5,6]. The internalin domain can be subdivided into an N-terminal helical cap domain, a leucine-rich repeat domain and an immunoglobulin-like inter-repeat (IR) domain. The function of most domains in InlB has been tested using N-terminal, C-terminal or internal deletions, or by expression of the isolated respective domains [4,7-14]. Using this approach the biochemical properties of the cap + LRR fragment, the IR domain, the B-repeat, the GW1 module and the GW2 + GW3 pair were addressed. These experiments showed that a cap + LRR fragment is sufficient for MET binding, but that the IR domain also contacts the receptor and contributes to MET activation [4,15]. The other domains (B-repeat, GW1-GW3) enhance MET activation by binding to co-receptors other than MET on host cells [7,10,11,14].

In the past, the cap and LRR domains have only been investigated together as a single unit, which appears reasonable from a structural point of view. In general, LRR proteins have specialized N- and C-terminal capping structures adjacent to the LRR domain that are thought to serve a structural role by shielding the hydrophobic core of the LRRs from solvent [16]. In InlB, the LRR and its flanking cap and IR domain share a single hydrophobic core, forming the internalin domain [17,18]. Deletion of the C-terminal capping structure, the IR domain, does not disturb the structure of the LRR, as InlB_241_ and InlB_248_, two different constructs lacking the IR domain, yield functional proteins [7,15,17]. Nevertheless the IR domain contributes significantly to the stability of InlB towards chemical and thermal denaturation [19]. To the best of our knowledge, biochemical experiments involving an internalin LRR domain lacking its N-terminal cap have not been reported. One would expect that deletion of the N-terminal cap would result in misfolding and aggregation or degradation of the LRR domain. This assumption is corroborated by the finding that InlB folds along a polarized pathway from the N- to the C-terminus [20]. An attempt to express a cap-less InlB in *E. coli* resulted in insoluble protein [21]. As a consequence, the function in receptor activation of the cap and the LRR domain of InlB has so far never been investigated individually.

## Results

### Generation and biochemical characterization of YopM-InlB

**Figure 1:**
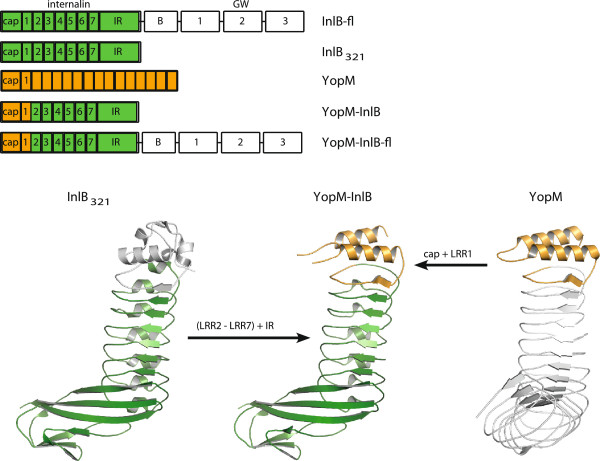
**Design strategy and crystal structure of YopM-InlB.** Domain organization of full-length InlB (InlB-fl), InlB_321_, YopM and the two hybrid proteins. LRRs are numbered in InlB but not in YopM. The structure of wild type InlB_321_ (PDB ID 1h6t) is shown on the bottom left. Residues 93–321 are highlighted in green. YopM (PDB ID 1jl5) is shown on the right. Residues 34–87 comprising the cap and LRR1 are highlighted in orange. The actual crystal structure of the hybrid protein YopM-InlB is shown in the middle.

To address a potential interaction of the InlB cap domain with a binding partner from the host cell, we sought to replace the cap by a structurally unrelated domain that still initiates folding. YopM is a leucine-rich repeat protein from *Yersinia enterocolitica* with 15 LRRs, each 20 or 22 residues in length [22]. The repeats in YopM are most similar to those of the internalins like InlB [22] and the overall horseshoe-shaped structure of YopM resembles that of the 15 LRR internalin InlA [23]. However, the N-terminal capping structures of *L. monocytogenes* internalins and *Y. eneterocolitica* YopM are structurally different. The cap domain of InlB resembles a truncated EF-hand motif consisting of three short helices and a two-stranded antiparallel β-sheet [17,18], while that of YopM consists of two longer α-helices connected by a single loop [22]. In InlB the cap is structurally closely linked to the first LRR. Residues 83 and 91 from LRR1 are alanine and valine instead of leucine or isoleucine in other LRRs. As a consequence, the hydrophobic core of LRR1 is loosely packed and the side chain of Phe53 from the cap fills the resulting hole. The capping domain of YopM does not provide a large side chain to fill the gap in LRR1 of InlB. We thus reasoned that replacing LRR1 along with the capping domain might increase protein stability. This, furthermore, allowed an EcoRI site in InlB to be used in cloning. The hybrid protein combines residues 34–87 of YopM with residues 93–321 of InlB and will be referred to as YopM-InlB (Figure 1). The protein was produced as GST-fusion and was purified like the InlB internalin domain (residues 36–321, referred to as InlB_321_) despite having a higher tendency to precipitate. Nevertheless, between 5 and 20 mg of pure YopM-InlB were obtained per litre of bacterial culture. The protein eluted as a single symmetric peak from gel filtration (see below). We used differential scanning fluorimetry (DSF) to assess the thermal stability of YopM-InlB and other InlB variants. With a melting temperature (T_M_) of 49,9°C (+/−0.22°C) YopM-InlB was destabilized by about 2 K with respect to InlB_321_ containing the native N-terminal cap (T_M_ of 51,9°C (+/− 0.21°C)). YopM-InlB is considerably more stable than InlB_241_ (T_M_ of 42,4°C (+/− 0.51°C)), a truncated form lacking the IR domain, which acts as C-terminal capping structure [15]. Two variants with an additional LRR inserted after the first LRR that bind and activate MET like wild type InlB_321_[21] showed melting temperatures close to that of InlB_321_ or YopM-InlB (T_M_ of InlB_321_ + 1LRRa was 52,5°C (+/−0.08°C); T_M_ of InlB_321_ + 1LRRb was 49,8°C (+/−0.16°C)). The results from DSF presented here are in good agreement with the endothermic denaturation transitions from differential scanning calorimetry reported previously for InlB_321_ (49.8°C) and for InlB_248_ (42.8°C) [19]. Small differences between the previous and our results may be due to different buffers or the different short InlB constructs (InlB_241_ vs. InlB_248_).

### Structure of YopM-InlB

We crystallized the YopM-InlB hybrid protein and solved its structure by molecular replacement. The numbering of both fragments in the PDB file corresponds to that of the proteins from which they were derived. The gap in numbers between the consecutive residues Pro87 and Gly93 represents the fusion point of Pro87 derived from YopM and directly adjacent Gly93 derived from InlB. No residues are actually missing in between.

**Figure 2:**
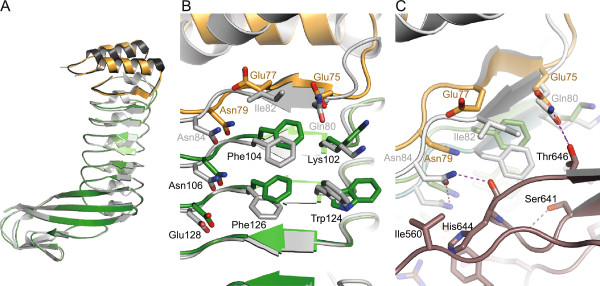
**Structural overlay of YopM-InlB with wild type InlB**_**321 **_**and YopM. (A)** YopM-InlB is colored orange and green as in Figure 1. Residues 34–87 from YopM (dark grey) and residues 93–321 from wild type InlB (light grey) are structurally aligned to highlight overall similarity and local differences. **(B)** YopM-InlB (colored as in Figure 1 and Figure 2A) was structurally aligned with InlB (grey). LRR1, which carries different sequences in the two proteins, shows the largest structural deviations. However, residues on the concave face of LRR2 and LRR3 are also shifted. **(C)** Overlay of YopM-InlB (colored as in Figure 1 and Figure 2A,B) onto InlB_321_ (grey) in the complex with MET (brown) (PDB ID 2uzx). Due to the shift in LRR1 residues Glu75 and Asn79 from YopM-InlB cannot form the hydrogen bonds to MET that are formed by the equivalent residues from wild type InlB (Gln80 and Asn84, respectively).

The structure of YopM-InlB at 1.5 Å resolution (Table 1) confirms that this designed chimeric protein is properly folded (Figure 1). The electron density is very well defined with exception of the N-terminal residues (up to Tyr39), two residues (Gly53, Asn54) in the loop connecting the two helices of the YopM cap domain and Glu95 and Tyr96, the first two residues of InlB directly after the fusion site. Structural alignment of the N-terminal cap structure and the first LRR of YopM (residues 34–87 of PDB ID 1jl5) with the hybrid protein results in an r.m.s.d. of 0.9 Å for 49 common Cα atoms (0.8 Å for 199 common main chain atoms; 1.3 Å for all 399 atoms). The largest deviations are located in the loop region connecting the two helices (Figure 2A). Structural alignment of InlB_321_ (PDB ID 1h6t) and the YopM-InlB hybrid protein for the region comprising residues 93–321 results in a coordinate r.m.s.d. of 1.0 Å for 223 common Cα atoms (1.0 Å for 901 common main chain atoms; 1.6 Å for all 1775 atoms). There are major shifts between wild type InlB and the hybrid protein in residues on the concave face of LRR1. For the three surface exposed residues (Gln80, Ile82, Asn84 in wild type InlB and Glu75, Glu77, Asn79 in YopM-InlB) the Cα atoms are shifted by 1.2 Å, 2.5 Å and 2.0 Å, respectively (Figure 2B). The side chains of InlB Gln80 and Asn84 form hydrogen bonds to Thr646 and His644 of MET, respectively, in the crystal structure of the InlB:MET complex [15]. Due to their relative shift, the equivalent atoms in the corresponding residues in YopM-InlB would no longer be able to form these hydrogen bonds (Figure 2C). Moreover, the structural differences between YopM-InlB and wild type InlB are not limited to LRR1, which was replaced by the YopM sequence. Instead rearrangements extend to LRR2 and to a lesser extent to LRR3. E.g. the Cα of Phe104, a residue located in the β-strand of LRR2 and essential for the binding of MET [24], is shifted by some 1.5 Å (Figure 2B). We had not anticipated this medium range effect, which can in retrospect be explained by a slightly lower curvature in the N-terminal region of the protein due to the absence of a 3_10_ helix in the first LRR of YopM. With only 20 residues, (two residues less than a typical internalin LRR) the first LRR of YopM is among the shortest LRRs known and it forms an extended rather than a helical structure on the convex face.

**Table 1:** Data collection and refinement statistics

**Data collection statistics**	
Beamline	DESY X13
Space group	*C*2
Unit cell dimensions [Å], [°]	a = 59.21
	b = 30.68
	c = 135.39
	β = 90.98
Resolution [Å]	20-1.50 (1.54-1.50)
Completeness [%]	96.3 (86.1)
Redundancy	3.8 (3.3)
Observations	141243 (6028)
Unique reflections	38095 (2490)
I/σ(I)	13.9 (1.8)
R-meas [%]	6.8 (82.4)
CC(1/2)	99.9 (64.8)
Molecules per asymm. unit	1
Solvent content [%]	35.6 (VM 1.91)
**Refinement statistics**	
R_work_ [%]	17.5 (30.3)
R_free_ [%]	21.9 (32.5)
Number of atoms total	2512
Protein/solvent/others	2262/250/0
R.m.s. deviation	
Bonds [Å]	0.021
Angles [°]	2.218
Ramachandran plot	
Residues in favored regions [%]	97.1
Residues in allowed regions [%]	2.9
Residues in disallowed regions [%]	0.0

### YopM-InlB has reduced affinity for MET

**Figure 3:**
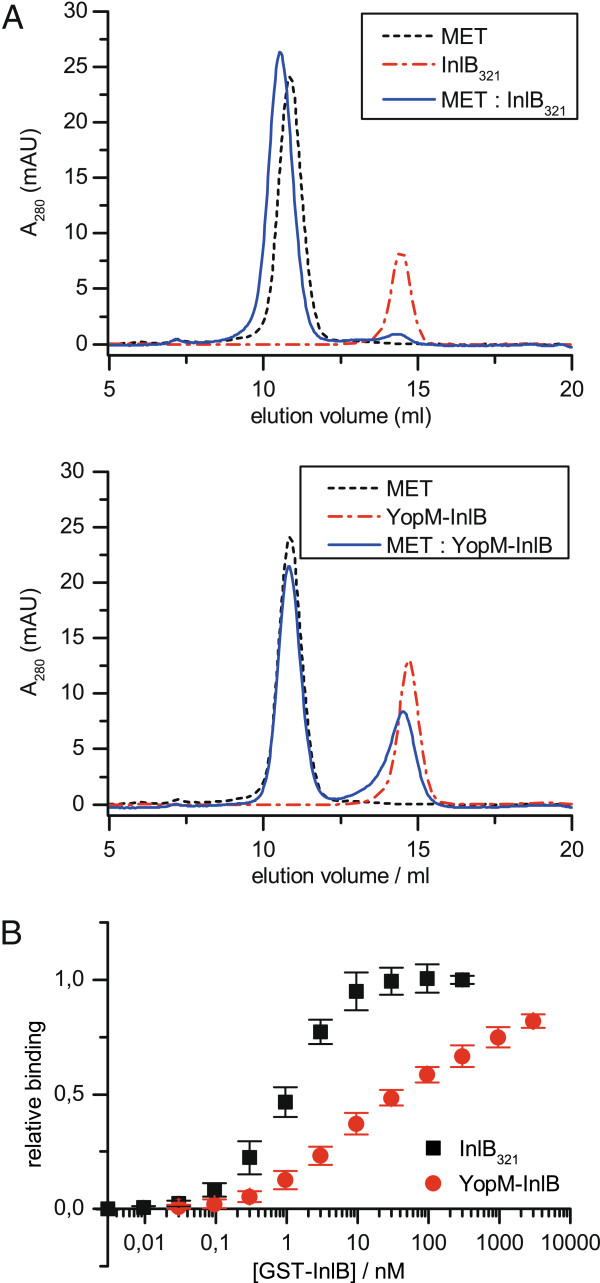
**Binding of YopM-InlB to MET. (A)** Elution profiles from gel filtration are shown for the complete MET ectodomain, for wild type InlB_321_, for YopM-InlB and the respective complexes. Wild type InlB_321_ is quantitatively shifted into a complex with MET eluting earlier than the isolated MET ectodomain. In contrast, the elution volume of YopM-InlB is only slightly shifted and its peak shows fronting indicating low affinity binding to MET, the elution behaviour of which is virtually unchanged. **(B)** Solid-phase binding assay, in which the complete MET ectodomain (MET_928_) was immobilized on ELISA plates and incubated with increasing concentrations of GST-tagged InlB_321_ and GST-tagged YopM-InlB. Binding was detected with a horseradish peroxidase-coupled anti-GST antibody. Three independent experiments, in which each protein was tested in triplicate, were normalized and then averaged. Error bars show standard deviation.

To assess whether the exchange of LRR1 and the unexpected structural changes in the β-sheet region of LRR2 and LRR3 impact MET binding, we investigated complex formation between the MET ectodomain and YopM-InlB by analytical gel filtration (Figure 3A, Table 2). We compared the elution profile of a stoichiometric 1:1 mixture of MET and YopM-InlB to that of MET and InlB_321_. Wild type InlB_321_ quantitatively shifted to lower elution volume indicating formation of a high affinity complex with the MET ectodomain, as observed previously [21,25]. In contrast, the peak for YopM-InlB did not disappear, but was broadened and became asymmetric (Figure 3A). The observed fronting and the shift to a slightly lower elution volume indicated weak binding of YopM-InlB to MET and separation of the complex during the gel filtration run.

**Table 2:** Elution volume from gel filtration and calculated molecular mass

	**Elution volume (ml)**	**Calculated M**_**r **_**(kDa)**
**MET**_**928**_	10.86	207.7
**InlB**_**321**_	14.46	36.7
**YopM-InlB**	14.71	32.5
**MET**_**928**_ **+ InlB**_**321**_	10.54	242.3
**MET**_**928**_ **+ YopM-InlB**	10.83/14.53	210.7/35.5

Next, we analysed the MET-binding ability of YopM-InlB in an enzyme-linked immunosorbent assay (ELISA) using immobilized MET ectodomain and soluble glutathione-S-transferase (GST)-fusion protein of the two InlB variants (Figure 3B). Binding affinity of YopM-InlB was strongly reduced compared to that of InlB_321_. The titration with YopM-InlB did not reach saturation at 3 μM, the highest concentration tested (Figure 3B). This is probably due to a high off-rate and perturbation of the binding equilibrium in the washing steps of the ELISA. Thus binding of YopM-InlB could not be quantitated reliably. However, our data from gel filtration and ELISA suggest that the binding affinity of YopM-InlB for MET may be several orders of magnitude weaker than that of wild type InlB, which has an affinity in the low nanomolar range [24,26].

### YopM-InlB is at least 10-fold less active in MET phosphorylation than wild type InlB_321_

**Figure 4:**
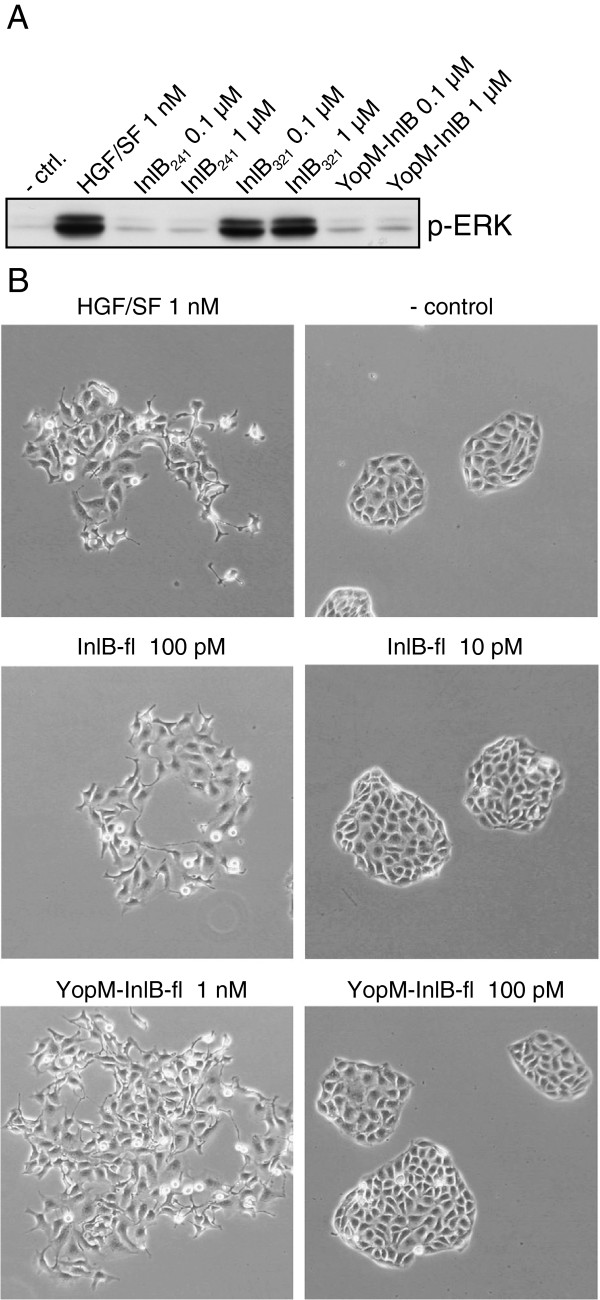
**Biological activity of YopM-InlB and of YopM-InlB-fl. (A)** The ability of the YopM-InlB to induce ERK phosphorylation was tested in Vero cells. Hepatocyte growth factor/scatter factor (HGF/SF) was used as positive control. InlB_241_ was used as negative control. The controls and reference in this figure (lane 1–6) are the same as those shown in a previously published experiment with InlB_321_ with an additional LRR inserted (Figure five c in [21]) as the experiments with both InlB cap variants (YopM-InlB and InlB_321_ + 1LRRa) were carried out in parallel. **(B)** The ability of YopM-InlB-fl to induce cell motility was tested in an MDCK cell colony scatter assay. HGF/SF was used as positive control, medium without ligand as negative control. YopM-InlB-fl stimulated colony dispersal at a concentration of 1 nM but showed no activity at 100 pM. The wild type protein was active at 10-fold lower concentration

InlB_321_ is sufficient to induce phosphorylation of MET and its downstream target ERK [7,15]. Here, we used ERK phosphorylation as readout for MET activation (Figure 4A). The shorter construct InlB_241_ that has the same affinity for MET as InlB_321_ but no biological activity [15] was used as negative control. InlB_321_ induced ERK phosphorylation in Vero cells at a concentration of 10^−7^ M. Like the negative control InlB_241_, YopM-InlB remained inactive even at a tenfold higher concentration. Given the reduced affinity of YopM-InlB for MET, this result is not unexpected. It is presumable due to the significantly lower affinity for MET.

### A YopM-InlB hybrid including the InlB C-terminal domains induces cell scatter though less efficiently than wild type InlB

Although InlB_321_ does stimulate phosphorylation of MET and ERK, it does not induce cell scatter, in contrast to full-length InlB (InlB-fl) [4,15,27]. To test whether the endogenous cap domain of InlB is essential for induction of a cellular response, we replaced the cap domain of InlB-fl by that of YopM (YopM-InlB-fl; Figure 1). As a readout, we used the well-established Madin Darbey canine kidney (MDCK) cell scatter assay (Figure 4B). YopM-InlB-fl induced the dispersal of MDCK cell colonies down to a concentration of 10^−9^ M but was inactive at 10^−10^ M, the lowest concentration at which wild type InlB-fl displayed activity. Thus, YopM-InlB-fl is about one order of magnitude less active than wild type InlB-fl.

## Discussion

### The internalin domain is a versatile framework allowing targeted manipulations

LRR proteins are promising targets for protein engineering and protein design because of their modular architecture. Synthetic libraries of designed LRR proteins have been used as artificial binders that might replace antibodies [28]. Some of the first structures of toll-like receptors (TLRs) were obtained with protein chimeras that combine the ligand-binding LRRs from TLRs with cap structures from variable lymphocyte receptors (VLRs) from hagfish [29]. The cap swap strategy presented here allows addressing the biological function of the N-terminal cap of internalins that cannot be studied with simple domain deletion constructs due to its contribution to protein folding. This approach is not limited to InlB but can similarly be applied to other internalins, as their cap and LRR domains are closely related. A potential biological function on top of that in protein folding could also be studied for the cap domain of the *Yersinia* protein YopM, by reversing the direction of the domain swap. In a related approach the cap domain of InlB along with LRR1 and LRR2 was fused to the ectodomain of VLRs in order to obtain proteins with favourable physicochemical properties [30].

### The InlB cap domain is not essential for MET activation

MET only interacts with the LRR and IR but not with the cap domain of InlB [15]. The reduced affinity of YopM-InlB for MET is presumably due to the exchange of the first LRR along with the cap domain resulting in spatial shifts of residues from LRR1, LRR2, and LRR3, whose side chains are involved in MET binding. The difference in affinity for MET between wild type and cap variant precludes a straightforward interpretation of the results from cellular assays. The failure of YopM-InlB to stimulate MET phosphorylation at a 10-fold higher concentration than required for InlB_321_ can presumably be ascribed to the reduced MET affinity. Still, the colony scatter experiments allow the conclusion that the cap is not essential for MET activation, although some contribution to receptor activation cannot be excluded conclusively from our experiments. Previous data had already shown that mutation of the potential calcium binding sites in the InlB cap domain has no appreciable effect on MET activation [31]. Our results extend this observation and show that, as long as a stable protein is formed, the cap domain as a whole can be replaced without causing a complete loss of activity.

Comparing the results of MET phosphorylation induced by YopM-InlB with cell scatter induced by YopM-InlB-fl shows that interaction of the C-terminal domains with their cellular receptors (i.e. heparan sulfate proteoglycans and/or gC1qR in the case of the GW domains [14,32,33] and an as of yet unidentified receptor for the B-repeat [11]) and the resulting avidity effect compensate the low affinity for the MET ectodomain at least partially. This resembles observations made with multiple arginine mutations preventing formation of an InlB dimer contact required for MET dimerization and activation. These mutations resulted in a more drastic effect in the isolated internalin domain (InlB_321_) than in InlB-fl [27]. One might conclude that interaction of various InlB domains with multiple host cell receptors endues InlB with a built-in redundancy that is able to offset a loss of affinity in one of the domains.

### Outlook

The modular design of LRR proteins does not only make them prime targets of rational protein design, but also renders them attractive model systems to investigate protein folding. The folding of both InlB [19,20,34] and YopM [35-37] has been studied extensively. Hence, fusion proteins like the one presented here that combine the N-terminal cap domain of one with the LRR domain of the other protein might represent new opportunities to address the importance of the N-cap for folding.

## Conclusions

The aim of this work was to replace the endogenous N-terminal cap structure of InlB by a structurally unrelated capping unit from another LRR protein in order to separate its structural role as folding nucleus from a potential role as binding site for an interaction partner. Conceptually, this aim was reached. The designed hybrid protein was folded and stable as shown by DSF, the single symmetric peak in gel filtration and the high-resolution crystal structure. In this particular case, the hybrid protein was not ideally suited for the intended functional studies, because it had reduced affinity for its receptor MET. Hence, the interpretation of results from cellular assays is not straightforward. However, our approach is not limited to InlB but can similarly be applied to other internalins and related LRR proteins.

## Methods

### Cloning of YopM-InlB

The cap domain together with the first LRR of *Yersinia enterocolitica* YopM was amplified from the 70 kilo base pair virulence plasmid pYVe227 with the primers aggag*c*|*catgg*gc**AAATCTAAGGCTGAATATTATAATGC** (forward) and aggag*g*|*aattc*c**CGGCAAAGAACTCAGCC** (reverse). The resulting PCR fragment contains the sequence for YopM amino acids Lys34 to Pro87 with two additional N-terminal residues (Met-Gly) due to the restriction site NcoI. The PCR fragment cleaved with NcoI and EcoRI was ligated into the vector pETM30-InlB_321_ (HN04-15) that had been cleaved with the same enzymes (removing amino acids 36 – 92 of InlB, which comprise the cap and most of LRR1) to yield the vector pETM30-YopM-InlB (HN06-01). The plasmid encoding YopM-InlB-fl was generated by excising an EcoRI/NotI fragment from the vector pGEX-6P-1-InlB-fl and cloning this into pETM30-YopM-InlB (HN06-01) to yield pETM30-YopM-InlB-fl (HN06-05).

### Protein expression and purification

All variants of InlB were produced as GST-fusions and purified essentially as described [15,21,27]. Briefly, *E. coli* BL21 (DE3) CodonPlus RIL cells were grown with shaking to an OD_600_ of 0.6 in LB medium at 37°C, shifted to 20°C, induced with 1 mM IPTG and further incubated over night. Harvested cells were resuspended in phosphate-buffered saline (PBS) with benzonase and complete protease inhibitor cocktail (Roche) and lysed in a French Press. After centrifugation the cleared lysate was applied to glutathione sepharose (GE Healthcare), followed by thorough washing with PBS. Fusion proteins used for ELISAs were eluted with reduced glutathione and further purified by anion exchange chromatography or size exclusion chromatography if necessary. Proteins used for assays with cells and for crystallization were cleaved from the GST-tag using tobacco etch virus (TEV) protease and further purified by cation exchange chromatography or size exclusion chromatography or both.

### Crystallization, data collection and structure determination

Initial crystals of YopM-InlB were obtained in sitting drops with a volume of 200 nl from Nextal screen MbClass in condition 42. Plate-shaped crystals for data collection were grown by hanging drop vapour diffusion at 20°C with a drop size of 2 μl consisting of equal volumes of protein at a concentration of 10 mg/ml and reservoir solution (0.1 M Tricine, pH 9.0, 28% PEG 1000, 10% glycerol, 0.25 M KCl). Crystals were cryo-protected with reservoir-solution additionally containing 15% glycerol and flash-frozen in liquid nitrogen. Data were collected on a MAR-CCD 165 detector at beamline X13, EMBL Hamburg, indexed and integrated with XDS, and scaled with XSCALE [38]. The phase problem was solved by molecular replacement using the program Phaser [39] with the appropriate fragments of InlB and YopM as search models. Errors were corrected and missing residues were added manually in the program Coot [40]. The structure was refined in the program REFMAC5 [41] using TLS groups suggested by the TLS motion detection server [42] and checked with MolProbity [43]. Data collection and refinement statistics are given in Table 1. Structural alignments were performed with LSQKAB [44]. Figures were prepared with PyMol [45]. Coordinates and structure factors were deposited in the PDB under accession code 4cil.

### Differential scanning fluorimetry

DSF was carried out essentially as described [46]. Proteins were measured at 0.1 mg/ml in PBS with a 1× SYPRO Orange concentration from 26°C to 95°C. The experiment was repeated four times with at least four data points for each protein and experiment. Data were analysed following a published protocol [47].

### Analytical gel filtration

40 μg of the complete MET ectodomain (MET_928_), equimolar amounts of wild type InlB_321_ or YopM-InlB or the respective mixtures were run on a Superdex200 10/300 GL column (GE Healthcare) equilibrated in PBS.

### Binding, phosphorylation and cell scatter assays

Binding, phosphorylation and cell scatter assays were carried out essentially as described [15]. The MET ectodomain (MET_928_) was purified from stably transfected CHO cells [48].

## Competing interests

The authors declare that they have no competing interest.

## Authors’ contributions

HHN initiated the study, cloned expression constructs, collected diffraction data and wrote the paper. DB and HHN purified protein, performed biochemical experiments and solved the structure. DB crystallized the protein. EG performed cell assays. WMB performed DSF analysis. All authors read and approved the final manuscript.
